# Supplementary material for: A QM/MM Study on the Initiation Reaction of Firefly Bioluminescence—Enzymatic Oxidation of Luciferin
Source: Molecules. 2021 Jul 12;26(14):4222. doi: 10.3390/molecules26144222 (PMC8307557; doi:10.3390/molecules26144222)
Supplement: Supplementary file 1 [file molecules-26-04222-s001.zip › molecules-1289981-supplementary.pdf]

Supporting Information

A QM/MM study on the initiation reaction of firefly bioluminescence - enzymatic oxidation of luciferin

Mohan Yu [1,2], Ya-Jun Liu[1,3,*]

[1] Center for Advanced Materials Research, Advanced Institute of Natural Sciences, Beijing Normal University at Zhuhai 519087, China.
[2] School of Chemistry and Chemical Engineering, Liaoning Normal University, Dalian 116029, China.
[3] Key Laboratory of Theoretical and Computational Photochemistry, Ministry of Education, College of Chemistry, Beijing Normal University, Beijing 100875, China.
* Correspondence: yajun.liu@bnu.edu.cn

Contents

1. Computational Details 2

1.1 PDB file 2

1.2 Ligand force field parameters 2

1.3 MM minization 2

Table S1 The protonation states of Histidine residue are computed by H++ program at pH =7.8. (His-245 is artificial definition) 3

1.4 MD simulation 3

Figure. S1. RMS deviation of firefly luciferase backbone during 2.5 ns molecular dynamics simulation. 4

1.5 QM/MM calculation 4

Figure. S2. QM/MM computational model. The colored ball and stick represents the QM region. The side chain of six residues are shown in grey stick. The line ribbon in the background represents the protein environment. . 5

2. Additional Figures 6

Figure. S3. The optimized structure of RC. (a) ground state structure (b)triplet state structure. 6

Figure. S4. (a) Computational model in DMSO and labels of key atoms. (b) S_0 and T_1 PECs of oxygenation of $\mathbf{A}^{3-}$ in DMSO at UM06-2X/6-31G (d, p) level. Red, yellow, black, blue and white balls represent oxygen, sulfur, carbon, nitrogen and hydrogen atoms. (The units of *ΔE* is kcal mol^{-1}) This work is published in Acta Chimica Sinica, 2020, 78(9):989-993 7

3. Additional Tables 8

Table S2 The spin densities of RC 8

4. Cartesian coordinates (in Å) 10

References 19

1. Computational Details

1.1 PDB file

To investigate firefly oxygenation, a firefly luciferase was chosen for suitable the oxygenation conformation. An empirical crystallographic structure the North American firefly *Photinus pyralis* luciferase PDB ID 4G37 with one molecule chain B in 4G37 structure [1]. Both two luciferase were downloaded from the Protein Data Pank [2].

1.2 Ligand force field parameters

In 4G37, the substrate DLSA 5'-O-[N-(dehydroluciferyl)-sulfamoyl] adenosine) was modified as FDO^- and AMP (adenosine 5'-monophosphate nucleotide). Due to O_2 molecule was hard handling in classic the molecular dynamics (MD) simulation, thus we modified DLSA as FDO^- and AMP. And then we assigned the atom types of Luc based on the standard AMBER atom type[3]. The charge parameters of above ligands were obtained using HF/6-31G (d) by the RESP fitting protocol [4]. This part calculated by Gaussian 16 [5]. The equilibrium bond lengths, bond angles, dihedral angles, force constants and van der Waals parameters for atom types similar to those defined by the GAFF parameter set [6].

1.3 MM minization

The AMBER Parm99SB force field was employed to model the residues of these protein. For Histidine residue, we computed their pK_a with the H++ program [7] to identify the protonation state of Histidine (His) residue, especially the His 245 was defined as HIP because of His 245 is as a base to receive proton (Table S1). The hydrogen atoms, the missing atoms, counter ions and water solvent were added by LEAP module of the AMBER package. The structure was immersed in an octahedral TIP3P water box [8] with a minimum solute wall distance of 10 **Å** using the LEAP

module. The systems were minimized before the simulation as follows: First, the heavy atoms were frozen, permitting the H atoms to move during a 4000-step minimization. Second, the protein, substrate were frozen and the solvent molecules were allowed to move during a 4000-step minimization.

Table S1 The protonation states of Histidine residue are computed by H++ program at pH =7.8. (His-245 is artificial definition)

27	46	76	171	212	221	244	245	310	332	419	431	461	489
HID	HID	HIE	HID	HIE	HIE	HIE	HIP	HIE	HIE	HIP	HIE	HIE	HIE

1.4 MD simulation

MD simulation was performed in the AMBER16 package [3]. The optimized system was heated stepwise from 0 to 300 K in 50 ps. The MD simulation was produced using the period boundary conditions at constant temperature T = 300 K and pressure P = 1 atm. The time step was 2 fs, and the system coordinates were recorded every 1 ps during the MD production. A default cutoff radius of 10 Å was introduced for nonbonding interactions. The electrostatic interactions were calculated by means of the Particle Mesh Ewald method [9]. The SHAKE algorithm [10] was applied to constrain all bond lengths involving hydrogen atoms. The coordinates of the simulated systems was collected every 1 ps during 2.5 ns MD production. As shown in Fig. S1, the equilibration is from 1600 ps to 2500 ps, and the initial structures for QM/MM calculation was started at the snapshot of 1800 ps from the MD trajectory.

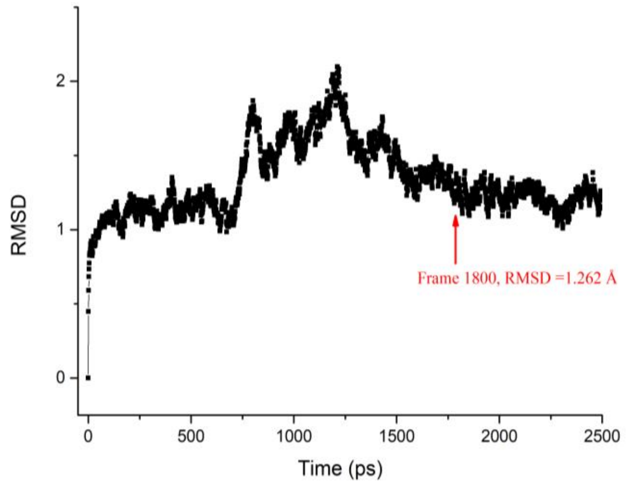

Figure. S1. RMS deviation of firefly luciferase backbone during 2.5 ns molecular dynamics simulation.

1.5 QM/MM calculation

To investigate the enzymatic oxidation in firefly luciferase, QM/MM calculations were performed based on the empirical crystallographic structure. An initial snapshot was chosen for QM/MM calculation according to the RMS deviation analysis. Our own N-layered integrated molecular orbital and molecular mechanics (ONIOM) was employed for QM/MM calculations. The protein, substrate, counter ions and water within 10 Å of substrate were using as the QM/MM model. For QM region, $\mathbf{L}^{3-}$ and O_2 was selected with a total of 59 atoms. While other atoms are in MM region, side chain of six residues (His-245, Gly-246, Phe-247, Gly-316, Gly-341, Thr-343) were allowed to relax (Fig. S2). In the QM/MM calculations, the UM06-2X/6-311G (d,p) method with BS technology was adopted for the QM region, while the Amber force field parm96 [11] to describe the MM protein environment. For the interaction between QM and MM region, the electronic embedding (EE) scheme was used which include the polarization effect of the MM region on the QM region. The unrestricted DFT was used to treat the reaction process including the open-shell singlet state and triplet state, while the broken-symmetry technology and spin projection method [12] were employed to

reduce the error caused by this method itself and obtain more accurate results. The Gaussian 16 program suite [5] was used for all QM/MM calculations.

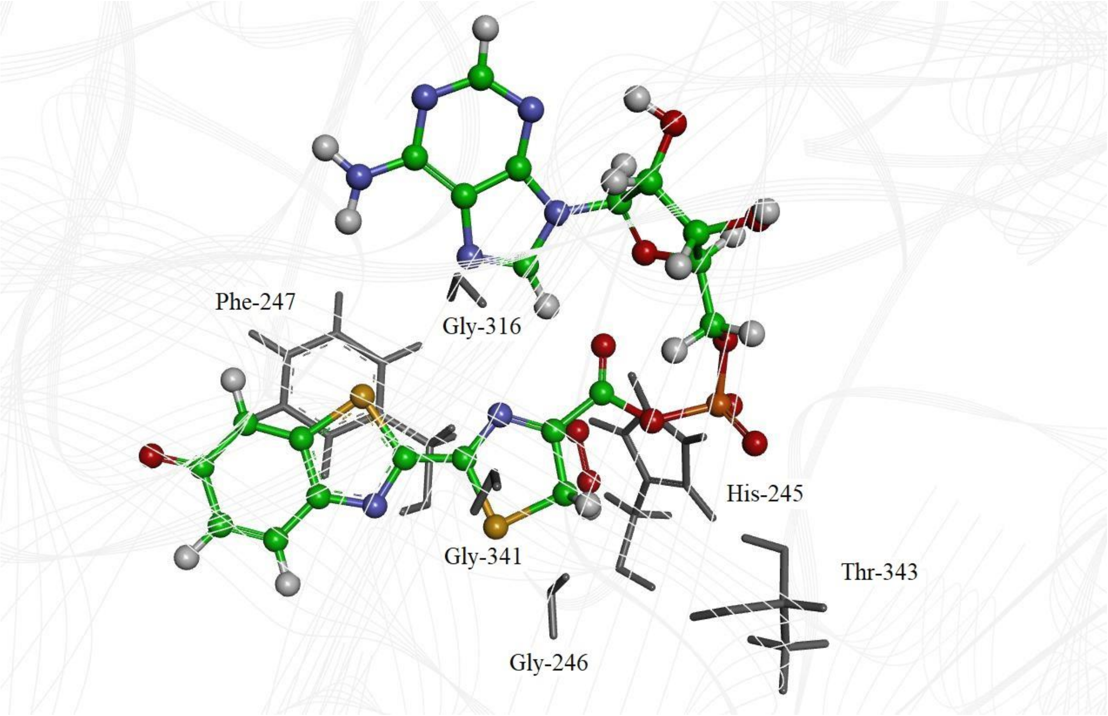

Figure. S2. QM/MM computational model. The colored ball and stick represents the QM region. The side chain of six residues are shown in grey stick. The line ribbon in the background represents the protein environment.

2. Additional Figures

(a) ^{1}RC (b) ^{3}RC

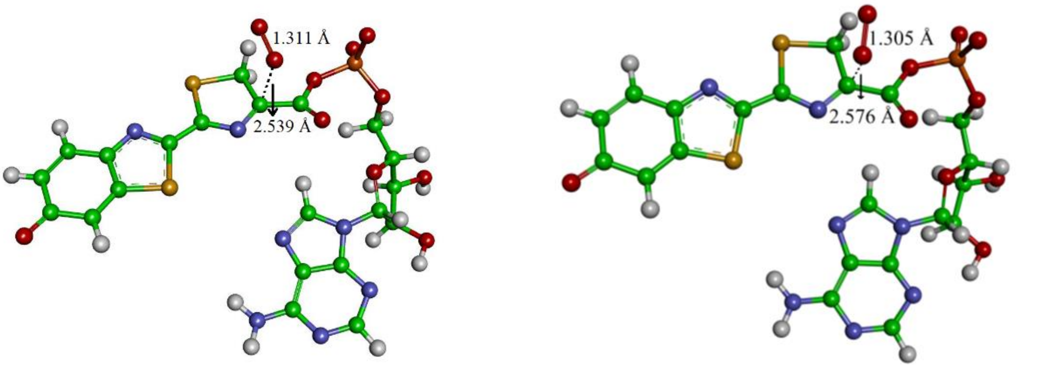

Figure. S3. The optimized structure of RC. (a) ground state structure (b)triplet state structure.

(a) Computational model in DMSO and labels of key atoms.

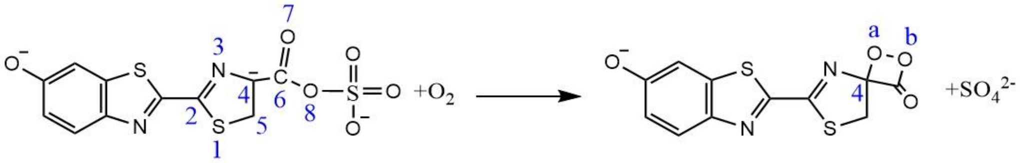

A^{3-} FDO$^-$

(b) PECs of oxygenation in DMSO

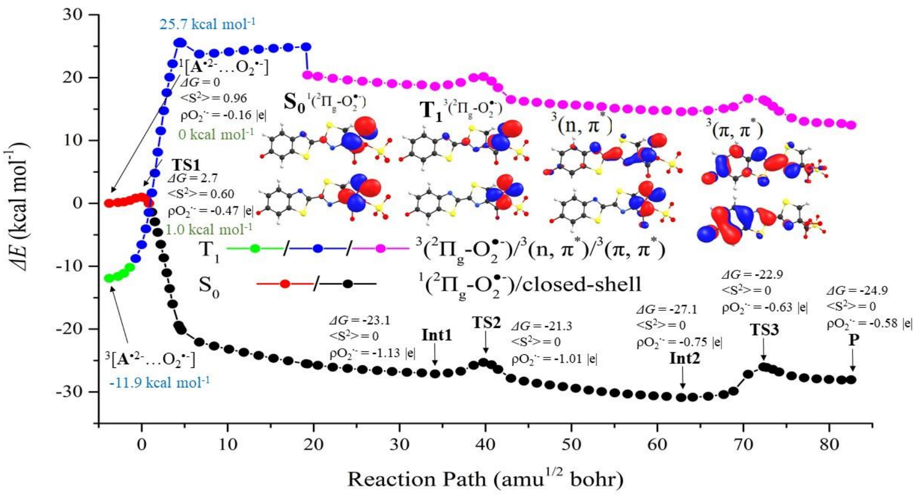

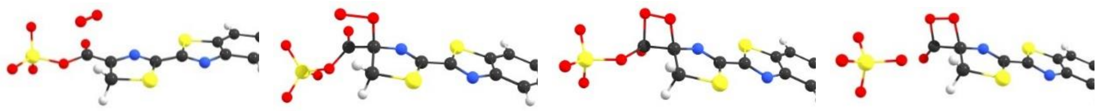

RC **Int1** **Int2** **P**

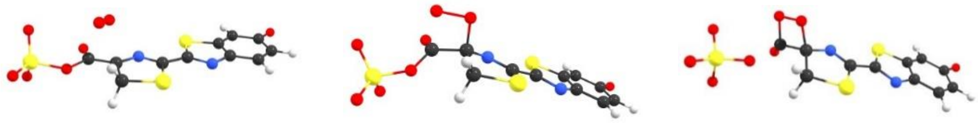

TS1 **TS2** **TS3**

Figure. S4. (a) Computational model in DMSO and labels of key atoms. (b) S_0 and T_1 PECs of oxygenation of $\mathbf{A}^{3-}$ in DMSO at UM06-2X/6-31G (d, p) level. Red, yellow, black, blue and white balls represent oxygen, sulfur, carbon, nitrogen and hydrogen atoms. (The units of ΔE is kcal mol^{-1}) This work is published in Acta Chimica Sinica, 2020, 78(9):989-993.

3. Additional Tables

Table S2 The spin densities of RC

Atom	spin densities $^1[L^{\bullet 2-}\ldots O_2^{\bullet -}]$	spin densities $^3[L^{\bullet 2-}\ldots O_2^{\bullet -}]$
O	0.464814	0.507798
O	0.479186	0.549103
C	-0.033740	0.029826
O	-0.089192	0.074480
C	-0.394090	0.380734
C	0.040126	-0.033593
S	-0.042315	0.043689
H	-0.017724	0.018062
H	-0.027642	0.015075
N	0.116698	-0.108149
C	-0.174930	0.189093
C	0.111506	-0.114244
N	-0.077130	0.085614
S	0.025122	-0.023773
C	-0.005892	0.007711
C	-0.097829	0.096928
C	0.021714	-0.019950
H	0.000013	-0.000125
C	-0.101496	0.102667
H	0.006125	-0.006221
C	0.000115	0.001107
O	-0.153591	0.156415
C	-0.048883	0.048459
H	0.003041	-0.003042
O	-0.001941	0.001784
P	-0.001788	0.000902
O	-0.000191	0.000007
O	-0.001023	0.000653
O	-0.000023	0.000022
C	-0.000291	0.000120

H	0.000351	-0.000419
H	-0.000047	0.000041
C	-0.000012	0.000051
O	-0.000129	0.000114
H	-0.000031	0.000025
C	-0.000017	0.000013
O	-0.000001	-0.000000
H	-0.000001	0.000001
H	-0.000025	0.000029
C	-0.000008	0.000005
O	-0.000004	0.000003
H	0.000000	-0.000000
H	-0.000005	0.000005
C	-0.000115	0.000108
H	-0.000000	0.000001
N	-0.000064	0.000027
C	0.000013	-0.000017
N	0.000008	-0.000006
C	-0.000005	0.000003
H	0.000002	-0.000001
N	0.000004	-0.000003
C	-0.000009	0.000006
N	0.000004	-0.000007
H	0.000000	-0.000000
H	-0.000022	0.000022
C	-0.000024	0.000031
N	0.000197	-0.000193
C	-0.001803	0.002099
H	0.002991	-0.003087

4. Cartesian coordinates (in Å)

The Cartesian coordinates was optimized equilibrium structures (only QM) reported in this paper.

S_0		RC		T_1		RC	
O	2.099095	-0.322017	-1.084828	O	2.088529	-0.252867	-1.070960
O	3.090899	-0.883996	-0.437198	O	2.787367	-1.094576	-0.359758
C	5.045827	-1.753100	-1.822205	C	5.025857	-1.723138	-1.826121
O	5.806745	-1.680894	-0.889627	O	5.731697	-1.591835	-0.852659
C	3.917466	-2.652484	-2.060236	C	3.926467	-2.639181	-2.077732
C	2.925117	-2.283460	-3.129858	C	2.927467	-2.274209	-3.144876
S	1.627020	-3.536968	-3.013363	S	1.612401	-3.505549	-2.991440
H	3.363542	-2.285410	-4.120545	H	3.352151	-2.303826	-4.141084
H	2.501647	-1.303507	-2.915745	H	2.520630	-1.286286	-2.944649
N	3.651707	-3.728027	-1.324484	N	3.663928	-3.718783	-1.339406
C	2.507718	-4.314251	-1.669973	C	2.516933	-4.297474	-1.673031
C	1.909328	-5.478228	-1.132089	C	1.922263	-5.469678	-1.139668
N	0.729094	-5.911168	-1.565906	N	0.739058	-5.896816	-1.565699
S	2.633094	-6.528659	0.099092	S	2.654865	-6.530046	0.076391
C	1.243096	-7.602148	0.003992	C	1.260905	-7.598744	-0.010607
C	0.353375	-7.062643	-0.982111	C	0.364689	-7.051473	-0.985951
C	-0.833847	-7.789915	-1.278401	C	-0.825996	-7.774957	-1.277102
H	-1.491267	-7.428784	-2.058171	H	-1.488344	-7.410532	-2.051074
C	-1.118994	-8.935080	-0.606847	C	-1.109055	-8.922212	-0.608465
H	-2.006076	-9.515314	-0.834360	H	-1.999320	-9.499054	-0.832219
C	-0.268353	-9.475466	0.452801	C	-0.252677	-9.469742	0.443283
O	-0.607333	-10.494456	1.082656	O	-0.591780	-10.48921	1.071985
C	0.966339	-8.749665	0.703525	C	0.986777	-8.749410	0.685815
H	1.630995	-9.144774	1.461889	H	1.656486	-9.149832	1.436886
O	5.178810	-0.875666	-2.920969	O	5.192247	-0.868446	-2.942220
P	6.082915	0.464676	-3.143995	P	6.082981	0.482151	-3.150035
O	6.054767	0.794032	-4.591399	O	6.048155	0.828704	-4.593124
O	5.614391	1.569666	-2.200942	O	5.608121	1.573167	-2.193150
O	7.559556	0.076247	-2.686670	O	7.564183	0.100876	-2.701934

C	8.194734	-0.995409	-3.386554	C	8.201450	-0.962930	-3.413497
H	7.472268	-1.786278	-3.616561	H	7.474995	-1.739995	-3.675111
H	8.643729	-0.618904	-4.306425	H	8.676514	-0.573089	-4.313634
C	9.252315	-1.535476	-2.491202	C	9.231074	-1.534723	-2.507754
O	8.656802	-2.213899	-1.416400	O	8.600364	-2.229548	-1.464168
H	9.940498	-0.767729	-2.148234	H	9.922187	-0.783374	-2.133224
C	10.203477	-2.461772	-3.205314	C	10.187280	-2.460596	-3.214033
O	11.135445	-1.569339	-3.756087	O	11.123250	-1.569063	-3.758892
H	11.610639	-2.005136	-4.456518	H	11.608751	-2.007916	-4.450381
H	9.671527	-3.030050	-3.973868	H	9.660791	-3.031942	-3.984105
C	10.633129	-3.431033	-2.077178	C	10.607075	-3.422987	-2.076748
O	11.908703	-3.202971	-1.546743	O	11.864400	-3.166316	-1.515733
H	12.015521	-3.970776	-0.964680	H	11.958227	-3.914612	-0.905962
H	10.557712	-4.465400	-2.403369	H	10.558859	-4.460045	-2.400108
C	9.547735	-3.195661	-0.984016	C	9.495494	-3.196437	-1.006682
H	10.055173	-2.907539	-0.056689	H	9.981937	-2.891155	-0.073284
N	8.787977	-4.389990	-0.723378	N	8.748336	-4.397224	-0.747717
C	9.370016	-5.474874	-0.139669	C	9.339159	-5.470458	-0.151261
N	10.624113	-5.558975	0.333468	N	10.592238	-5.537389	0.327607
C	10.820544	-6.743409	0.918250	C	10.800568	-6.717189	0.918328
H	11.802626	-6.912479	1.347386	H	11.783318	-6.872429	1.351108
N	9.961264	-7.751642	1.044836	N	9.953164	-7.735167	1.046745
C	8.741831	-7.660390	0.478123	C	8.733256	-7.658864	0.478114
N	7.926288	-8.699392	0.530685	N	7.927752	-8.705057	0.532897
H	8.114657	-9.412527	1.223051	H	8.120666	-9.413913	1.228394
H	6.981275	-8.686149	0.163101	H	6.982440	-8.699516	0.165240
C	8.391919	-6.451409	-0.151423	C	8.371353	-6.456658	-0.157864
N	7.233318	-5.988963	-0.743814	N	7.210053	-6.010744	-0.757487
C	7.508594	-4.754098	-1.077380	C	7.473720	-4.776691	-1.102235
H	6.833038	-4.054240	-1.547133	H	6.793849	-4.090827	-1.586652

S_0		**Int1**		S_0		**TS1**	
O	2.354294	-0.302749	-1.078497	O	2.169159	-0.466969	-1.214913
O	2.555471	-1.653103	-0.634226	O	3.195126	-1.126677	-0.578303

C	4.658791	-1.563993	-1.619050	C	4.995503	-1.714235	-1.770145
O	5.296113	-1.341213	-0.626934	O	5.836106	-1.734962	-0.911941
C	3.296842	-2.341355	-1.602978	C	3.701820	-2.458491	-1.819851
C	2.620127	-2.322193	-2.970920	C	2.770594	-2.192976	-2.893433
S	1.327682	-3.571212	-2.818269	S	1.628302	-3.548116	-2.950435
H	3.314072	-2.596427	-3.760282	H	3.151246	-1.863181	-3.848544
H	2.198491	-1.339983	-3.150213	H	2.206428	-1.211949	-2.268125
N	3.339021	-3.723067	-1.105062	N	3.516583	-3.657678	-1.136363
C	2.295363	-4.350968	-1.520585	C	2.450831	-4.271664	-1.567837
C	1.735373	-5.623278	-1.071630	C	1.840389	-5.481771	-1.054769
N	0.729094	-6.036301	-1.533788	N	0.704581	-5.927202	-1.536440
S	2.633094	-6.699969	0.149280	S	2.527045	-6.498948	0.211373
C	1.243096	-7.774189	0.003295	C	1.163218	-7.599850	0.066517
C	0.353375	-7.206288	-0.983914	C	0.309436	-7.084980	-0.954828
C	-0.833847	-7.911817	-1.315284	C	-0.855072	-7.827445	-1.283321
H	-1.491267	-7.522479	-2.091547	H	-1.493428	-7.481647	-2.086461
C	-1.118994	-9.072895	-0.678538	C	-1.147119	-8.971564	-0.608954
H	-2.006076	-9.635791	-0.940431	H	-2.019369	-9.563960	-0.862256
C	-0.268353	-9.654419	0.385376	C	-0.329513	-9.495843	0.485201
O	-0.607333	-10.687525	0.983840	O	-0.677249	-10.51547	1.112659
C	0.966339	-8.944824	0.672568	C	0.881308	-8.750440	0.768733
H	1.630995	-9.357223	1.434303	H	1.527316	-9.123452	1.553853
O	5.178810	-1.050962	-2.837859	O	5.124818	-0.891380	-2.883425
P	6.082915	0.221320	-3.120229	P	6.089672	0.408634	-3.136562
O	6.054767	0.467592	-4.580028	O	6.085300	0.702169	-4.589127
O	5.614391	1.395178	-2.246555	O	5.631080	1.538709	-2.219333
O	7.559556	-0.294659	-2.582304	O	7.532800	-0.039993	-2.638503

C	8.194734	-1.281129	-3.364781	C	8.145781	-1.159479	-3.288685
H	7.472268	-2.193548	-3.433012	H	7.439674	-1.993859	-3.355051
H	8.643729	-0.886303	-4.368434	H	8.444983	-0.857117	-4.295218
C	9.252315	-1.511639	-2.630271	C	9.334105	-1.518485	-2.448798
O	8.656802	-2.036877	-1.339585	O	8.903533	-2.104939	-1.220444
H	9.940498	-0.555791	-2.495150	H	9.954012	-0.658943	-2.259007
C	10.203477	-2.465685	-3.320855	C	10.312273	-2.458916	-3.100783
O	11.135445	-1.902477	-3.231082	O	11.578867	-2.147193	-2.573902
H	11.610639	-2.546900	-3.700001	H	12.048051	-2.988224	-2.595204
H	9.671527	-2.652845	-4.364699	H	10.239126	-2.355153	-4.175788
C	10.633129	-3.742130	-2.491429	C	9.867284	-3.784046	-2.539070
O	11.908703	-4.517993	-2.430291	O	10.869075	-4.761753	-2.623259
H	12.015521	-4.949156	-1.567642	H	10.959332	-5.215653	-1.775835
H	10.557712	-4.309680	-2.911800	H	8.930524	-4.096661	-3.015835
C	9.547735	-3.231312	-1.123170	C	9.533114	-3.353498	-1.107635
H	10.055173	-3.097384	-0.449027	H	10.436994	-3.296064	-0.495469
N	8.787977	-4.221134	-0.523301	N	8.683489	-4.341565	-0.501712
C	9.370016	-5.364978	0.016800	C	9.278961	-5.449815	0.050632
N	10.624113	-5.539643	0.453929	N	10.542716	-5.550376	0.489203
C	10.820544	-6.761064	0.970672	C	10.772936	-6.762824	0.997688
H	11.802626	-6.993768	1.364436	H	11.768581	-6.938409	1.391325
N	9.961264	-7.736393	1.062161	N	9.933924	-7.787979	1.081327
C	8.741831	-7.568226	0.514409	C	8.707151	-7.684431	0.534046
N	7.926288	-8.592883	0.509936	N	7.925038	-8.744764	0.518986
H	8.114657	-9.357139	1.144053	H	8.164938	-9.511820	1.132571
H	6.981275	-8.559775	0.150291	H	6.966202	-8.730968	0.188330
C	8.391919	-6.307629	-0.041057	C	8.327844	-6.446309	-0.019103

N	7.233318	-5.785650	-0.643637	N	7.176461	-5.993947	-0.637894
C	7.508594	-4.556891	-0.934651	C	7.433360	-4.749486	-0.930527
H	6.833038	-3.835039	-1.417304	H	6.752253	-4.058772	-1.407750

S_0		Int2		S_0		TS2	
O	4.035508	-0.576574	-0.953200	O	3.121504	-0.569705	-0.970870
O	3.030180	-1.593031	-0.739608	O	2.963810	-1.964072	-0.646216
C	4.950000	-1.616525	-1.658998	C	4.938196	-1.772804	-1.770175
O	5.949532	-1.934364	-1.012276	O	5.730239	-1.748755	-0.871292
C	3.596352	-2.460903	-1.754468	C	3.579180	-2.569781	-1.737821
C	2.812733	-2.300646	-3.054670	C	2.747603	-2.351754	-3.008135
S	1.403794	-3.426198	-2.886970	S	1.404049	-3.543824	-2.844140
H	3.417499	-2.577146	-3.913082	H	3.312047	-2.537933	-3.917597
H	2.439357	-1.288345	-3.167440	H	2.348173	-1.345102	-2.969855
N	3.486897	-3.824165	-1.296881	N	3.600625	-4.001523	-1.429027
C	2.379373	-4.351154	-1.684975	C	2.468320	-4.510189	-1.758993
C	1.788481	-5.612295	-1.291689	C	1.877206	-5.779218	-1.370194
N	0.551594	-5.922919	-1.614976	N	0.622738	-6.043189	-1.646727
S	2.631256	-6.864208	-0.374438	S	2.703008	-7.058377	-0.474108
C	1.191391	-7.874857	-0.444133	C	1.223097	-8.017930	-0.495130
C	0.193307	-7.146712	-1.165586	C	0.232185	-7.254884	-1.183774
C	-1.069292	-7.769030	-1.359276	C	-1.054598	-7.829381	-1.345912
H	-1.848830	-7.249914	-1.899216	H	-1.825843	-7.277664	-1.867208
C	-1.298144	-9.016466	-0.876793	C	-1.313732	-9.073039	-0.863691
H	-2.252094	-9.508408	-1.030983	H	-2.287358	-9.531932	-0.995441
C	-0.305811	-9.791791	-0.130559	C	-0.328527	-9.887201	-0.153815

O	-0.584489	-10.92548	0.297904	O	-0.629396	-11.01970	0.269898
C	0.977543	-9.137703	0.059717	C	0.979050	-9.277036	0.008543
H	1.737461	-9.675749	0.612828	H	1.734541	-9.844724	0.537559
O	5.208142	-0.964366	-2.976454	O	5.179582	-1.096309	-2.955181
P	6.167731	0.314153	-3.196097	P	6.174106	0.201142	-3.185346
O	6.134187	0.645909	-4.648010	O	6.177015	0.458951	-4.645557
O	5.755721	1.476386	-2.281533	O	5.751255	1.367935	-2.304662
O	7.622039	-0.108085	-2.695216	O	7.597610	-0.232903	-2.632376
C	8.201973	-1.306082	-3.213518	C	8.298040	-1.300789	-3.262982
H	7.491744	-2.133193	-3.130841	H	7.652747	-2.181394	-3.354064
H	8.458762	-1.143849	-4.264610	H	8.610959	-0.980751	-4.260310
C	9.431591	-1.548729	-2.384549	C	9.475627	-1.564559	-2.371777
O	9.085588	-2.082036	-1.105584	O	9.028384	-2.125880	-1.137814
H	9.995579	-0.641834	-2.266240	H	10.041207	-0.666683	-2.194954
C	10.443689	-2.467459	-3.005088	C	10.497247	-2.484723	-2.968985
O	11.705043	-2.123155	-2.480676	O	11.749526	-2.167937	-2.411381
H	12.174066	-2.965199	-2.489307	H	12.210388	-3.014589	-2.439417
H	10.367082	-2.374879	-4.078204	H	10.443588	-2.370770	-4.040745
C	10.033185	-3.790487	-2.419287	C	10.050240	-3.811153	-2.413746
O	11.061866	-4.743795	-2.477693	O	11.066303	-4.776209	-2.449571
H	11.133444	-5.200108	-1.629698	H	11.138068	-5.211513	-1.590012
H	9.106660	-4.135577	-2.891621	H	9.134770	-4.137585	-2.919006
C	9.682822	-3.342463	-0.994031	C	9.650770	-3.374035	-1.000108
H	10.574516	-3.308680	-0.362630	H	10.522696	-3.319195	-0.343297
N	8.800064	-4.323754	-0.414778	N	8.767125	-4.361407	-0.442392
C	9.365610	-5.460210	0.103907	C	9.336854	-5.484469	0.100917
N	10.623714	-5.600600	0.550384	N	10.594223	-5.605353	0.553587

C	10.821080	-6.827943	1.031436	C	10.803259	-6.828591	1.042759
H	11.810403	-7.040655	1.422510	H	11.793911	-7.028438	1.436965
N	9.952157	-7.829735	1.091395	N	9.945153	-7.837936	1.105348
C	8.734385	-7.686997	0.536129	C	8.726871	-7.713626	0.546066
N	7.924771	-8.729105	0.512054	N	7.927320	-8.759432	0.523858
H	8.146378	-9.504695	1.121311	H	8.155039	-9.534931	1.131443
H	6.971764	-8.697270	0.169419	H	6.974328	-8.735405	0.178178
C	8.393676	-6.434062	-0.005299	C	8.376107	-6.467683	-0.005508
N	7.260080	-5.938134	-0.623609	N	7.246877	-5.994146	-0.646355
C	7.546493	-4.687377	-0.873316	C	7.526406	-4.748581	-0.914996
H	6.885220	-3.947869	-1.311169	H	6.864984	-4.044947	-1.400645

S_0		**P**		S_0		**TS3**	
O	46.167323	46.211612	44.081036	O	4.132743	-0.83785	-0.700850
O	46.994154	45.166469	44.639455	O	2.845479	-1.41428	-1.024730
C	45.679202	45.382947	43.098463	C	4.832527	-1.86142	-1.384995
O	44.932394	45.722835	42.253767	O	5.901469	-2.23439	-1.036457
C	46.621980	44.252864	43.518134	C	3.479758	-2.50433	-1.792043
C	46.129547	42.920004	44.043420	C	2.944690	-2.47830	-3.201164
S	47.605388	41.854865	43.979835	S	1.435794	-3.48996	-3.085537
H	45.341896	42.533794	43.406201	H	3.655332	-2.93156	-3.875922
H	45.751966	43.009622	45.050890	H	2.715204	-1.47962	-3.546120
N	47.717942	44.117764	42.630418	N	3.235399	-3.77878	-1.183144
C	48.352129	43.013396	42.853540	C	2.181342	-4.33186	-1.688674
C	49.627559	42.695875	42.301749	C	1.571606	-5.56525	-1.269957
N	50.365855	41.698263	42.730326	N	0.360806	-5.93187	-1.644842

S	50.407570	43.754440	41.127042	S	2.413702	-6.75894	-0.275670
C	51.845781	42.766800	41.235484	C	1.023920	-7.82676	-0.396150
C	51.590663	41.709722	42.172993	C	0.027582	-7.15125	-1.176305
C	52.640767	40.787988	42.438408	C	-1.208242	-7.82239	-1.397993
H	52.454422	39.968567	43.122767	H	-1.990129	-7.33427	-1.961254
C	53.841555	40.942552	41.823113	C	-1.405460	-9.06825	-0.903218
H	54.656250	40.249567	41.989158	H	-2.335153	-9.59822	-1.076982
C	54.137200	42.029631	40.882794	C	-0.408018	-9.79396	-0.111898
O	55.267476	42.161794	40.384336	O	-0.655388	-10.9310	0.320682
C	53.044922	42.929030	40.593578	C	0.839686	-9.08634	0.121193
H	53.232027	43.738353	39.898969	H	1.596387	-9.58550	0.713593
O	43.817630	44.305893	44.588998	O	5.243513	-0.75175	-3.066291
P	42.396482	44.827824	44.549398	P	6.120993	0.51256	-3.212411
O	41.305900	43.805430	44.873760	O	6.105665	1.04858	-4.617775
O	42.243732	46.123751	45.380181	O	5.725135	1.62871	-2.201403
O	42.048123	45.380385	43.023675	O	7.626955	0.10313	-2.752589
C	42.125206	44.507133	41.902941	C	8.176089	-1.06653	-3.341779
H	43.146902	44.137233	41.779619	H	7.423497	-1.86085	-3.382215
H	41.444629	43.657364	42.037165	H	8.506049	-0.83265	-4.359433
C	41.692296	45.270518	40.666372	C	9.345091	-1.48092	-2.493664
O	42.666957	46.292942	40.389979	O	8.899189	-2.13921	-1.299390
H	40.720831	45.743284	40.849247	H	9.972951	-0.64800	-2.234043
C	41.584014	44.453902	39.366311	C	10.334525	-2.39092	-3.172586
O	40.322535	43.834197	39.232002	O	11.588081	-2.14655	-2.574528
H	40.167965	43.616313	38.272430	H	12.031549	-3.00019	-2.608606
H	42.392285	43.709593	39.330699	H	10.310660	-2.19615	-4.236339
C	41.841009	45.553241	38.316374	C	9.837846	-3.73945	-2.723166

O	40.678339	46.314348	38.106967	O	10.803283	-4.75103	-2.862907
H	40.235399	45.939815	37.298123	H	10.830555	-5.28598	-2.061418
H	42.237735	45.174702	37.374623	H	8.894999	-3.97890	-3.228765
C	42.885255	46.429053	39.009270	C	9.522490	-3.39258	-1.265971
H	42.770440	47.465184	38.686729	H	10.439361	-3.37233	-0.670484
N	44.258681	46.046431	38.665502	N	8.693850	-4.41209	-0.684429
C	44.986106	46.600230	37.636297	C	9.277998	-5.47328	-0.037565
N	44.623218	47.601367	36.826956	N	10.524030	-5.54112	0.452671
C	45.600030	47.907661	35.976597	C	10.748596	-6.72547	1.020925
H	45.385031	48.701475	35.268232	H	11.733686	-6.87212	1.450804
N	46.818997	47.371525	35.874404	N	9.918778	-7.76006	1.122676
C	47.160918	46.361725	36.693761	C	8.706684	-7.68610	0.543205
N	48.410989	45.881889	36.611689	N	7.928319	-8.75313	0.540595
H	49.083486	46.379583	36.035461	H	8.137642	-9.48600	1.205015
H	48.709935	44.988589	36.978743	H	6.982818	-8.74842	0.175478
C	46.196822	45.910615	37.627780	C	8.329863	-6.47636	-0.066051
N	46.217715	44.947919	38.621328	N	7.191261	-6.06994	-0.737183
C	45.055695	45.064599	39.210477	C	7.457818	-4.84830	-1.113623
H	44.733004	44.497506	40.069241	H	6.802330	-4.19108	-1.665622

References

1. Sundlov, J. A.; Fontaine, D. M.; Southworth, T. L.; Branchini, B. R.; Gulick, A. M., Crystal Structure of Firefly Luciferase in a Second Catalytic Conformation Supports a Domain Alternation Mechanism. *Biochem.* **2012,** 51, 6493-6495.
2. http://www.rcsb.org/.
3. D.A. Case, R. M. B., D.S. Cerutti, T.E. Cheatham, III, T.A. Darden, R.E. Duke, T.J. Giese, H. Gohlke, A.W. Goetz, N. Homeyer, S. Izadi, P. Janowski, J. Kaus, A. Kovalenko, T.S. Lee, S. LeGrand, P. Li, C. Lin, T. Luchko, R. Luo, B. Madej, D. Mermelstein, K.M. Merz, G. Monard, H. Nguyen, H.T. Nguyen, I. Omelyan, A. Onufriev, D.R. Roe, A. Roitberg, C. Sagui, C.L. Simmerling, W.M. Botello-Smith, J. Swails, R.C. Walker, J. Wang, R.M. Wolf, X. Wu, L. Xiao and P.A. Kollman, AMBER 2016. *University of California, San Francisco.* **2016**.
4. Cieplak, P.; Cornell, W. D.; Bayly, C.; Kollman, P. A., Application of the multimolecule and multiconformational RESP methodology to biopolymers: charge derivation for DNA, RNA, and proteins. *J. Comput. Chem.* **1995,** 16, 1357-77.
5. M. J. Frisch, G. W. T., H. B. Schlegel, G. E. Scuseria, M. A. Robb, J. R. Cheeseman, G. Scalmani, V. Barone, G. A. Petersson, H. Nakatsuji, X. Li, M. Caricato, A. V. Marenich, J. Bloino, B. G. Janesko, R. Gomperts, B. Mennucci, H. P. Hratchian, J. V. Ortiz, A. F. Izmaylov, J. L. Sonnenberg, D. Williams-Young, F. Ding, F. Lipparini, F. Egidi, J. Goings, B. Peng, A. Petrone, T. Henderson, D. Ranasinghe, V. G. Zakrzewski, J. Gao, N. Rega, G. Zheng, W. Liang, M. Hada, M. Ehara, K. Toyota, R. Fukuda, J. Hasegawa, M. Ishida, T. Nakajima, Y. Honda, O. Kitao, H. Nakai, T. Vreven, K. Throssell, J. A. Montgomery, Jr., J. E. Peralta, F. Ogliaro, M. J. Bearpark, J. J. Heyd, E. N. Brothers, K. N. Kudin, V. N. Staroverov, T. A. Keith, R. Kobayashi, J. Normand, K. Raghavachari, A. P. Rendell, J. C. Burant, S. S. Iyengar, J. Tomasi, M. Cossi, J. M. Millam, M. Klene, C. Adamo, R. Cammi, J. W. Ochterski, R. L. Martin, K. Morokuma, O. Farkas, J. B. Foresman, D. J. Fox, Gaussian 16. *Gaussian, Inc.* **2016**.
6. Wang, J. M.; Wolf, R. M.; Caldwell, J. W.; Kollman, P. A.; Case, D. A., Development and testing of a general amber force field. *J. Comput. Chem.* **2004,** 25, 1157-1174.
7. http://biophysics.cs.vt.edu/H++. http://biophysics.cs.vt.edu/H++.
8. Jorgensen, W. L.; Chandrasekhar, J.; Madura, J. D.; Impey, R. W.; Klein, M. L., Comparison of simple potential functions for simulating liquid water. *J. Chem. Phys.* **1983,** 79, 926-35.
9. Darden, T.; York, D.; Pedersen, L., Particle mesh Ewald: an N·log(N) method for Ewald sums in large systems. *J. Chem. Phys.* **1993,** 98, 10089-92.
10. Ryckaert, J. P.; Ciccotti, G.; Berendsen, H. J. C., Numerical integration of the Cartesian equations of motion of a system with constraints: molecular dynamics of n-alkanes. *J. Comput. Phys.* **1977,** 23, 327-41.
11. Cornell, W. D.; Cieplak, P.; Bayly, C. I.; Gould, I. R.; Merz, K. M.; Ferguson, D. M.; Spellmeyer, D. C.; Fox, T.; Caldwell, J. W.; Kollman, P. A., A 2ND GENERATION FORCE-FIELD FOR THE SIMULATION OF PROTEINS, NUCLEIC-ACIDS, AND ORGANIC-MOLECULES. *Journal Of the American Chemical Society* **1995,** 117, 5179-5197.
12. Yamaguchi, K.; Jensen, F.; Dorigo, A.; Houk, K. N., A SPIN CORRECTION PROCEDURE FOR UNRESTRICTED HARTREE-FOCK AND MOLLER-PLESSET WAVEFUNCTIONS FOR SINGLET DIRADICALS AND POLYRADICALS. *Chemical Physics Letters* **1988,** 149, 537-542.
